# Powder Surface Roughness as Proxy for Bed Density in Powder Bed Fusion of Polymers

**DOI:** 10.3390/polym14010081

**Published:** 2021-12-26

**Authors:** Francesco Sillani, Ramis Schiegg, Manfred Schmid, Eric MacDonald, Konrad Wegener

**Affiliations:** 1 Inspire, Innovation Center for Additive Manufacturing Switzerland (ICAMS), Fürstenlandstrasse 122, 9014 St. Gallen, Switzerland; manfred.schmid@inspire.ethz.ch; 2 Swiss Federal Institute of Technology, Institute of Machine Tools and Manufacturing (IWF), ETH Zurich, 8092 Zurich, Switzerland; schieggr@student.ethz.ch (R.S.); wegener@iwf.mavt.ethz.ch (K.W.); 3 University of Texas, El Paso, TX 79902, USA; emac@utep.edu

**Keywords:** polymer powder bed fusion, laser profilometry, powder layer density, roughness, powder layer topography

## Abstract

Powder bed fusion of polymers is becoming increasingly adopted by a variety of industries to tailor the strength, weight and functionality of end-use products. To meet the high standards of the modern manufacturing industry, parts built with powder bed fusion require consistent properties and to be free of defects, which is intrinsically connected to the quality of the powder bed prior to melting. The hypothesis of this work is that the roughness of the top surface of an unmelted powder bed can serve as a proxy for the powder bed density, which is known to correlate with final part density. In this study, a laser line scan profilometer is integrated onto the recoater arm of a custom powder test bench, which is able to automatically create layers of powder. A diverse group of polymers was investigated including polyamide 12 (PA12), polyamide 11 (PA11), polypropylene (PP), and a thermoplastic elastomer (TPU) under different recoating speed in order to increase the variance of the dataset. Data analytics were employed to compare roughness to measured powder bed density and a statically significant correlation was established between them.

## 1. Introduction

Laser powder bed fusion is the most industrialized additive manufacturing technology available on the market. Utilizing polymer or metal feedstock in powder form, complex geometries are fabricated striking the balance of mechanical performance versus weight in a layer-by-layer manner, even without support structures in the case of polymer powders. Nevertheless, for this next generation of manufacturing technology to be more widely adopted in industrial applications, quality assurance will be crucial as material properties and part geometry are realized simultaneously. Consequently, proper in situ measurements are required to provide insights on the quality of the produced parts by ensuring the process is within specification. Layer wise monitoring enables a qualify-as-you-go methodology improving yield, quality and consistency among parts produced within the same or even different build jobs. Due to the intrinsic nature of the layer-by-layer processing powder bed additive manufacturing, which exposes the top surface of the part during intermediate stages of fabrication, a series of layerwise digital scans can be captured as shown by many authors [1,2,3,4,5]. However, process monitoring in polymer powder bed fusion is generally absent in industrial-grade commercial systems and remains an expensive yet unproven option even in metal laser powder bed fusion, for example with the optical tomography (OT) monitoring systems (with either visible or infrared light) provided with either visible or infrared recording such as the EOS EOSTATE Exposure OT [6]. As reported by Mussatto et al. [7], powder morphology, spreading conditions and the interaction of the particles influence powder bed topography, and error-free spreading is crucial in order to minimize process failures such as short-feeds or voids such as the ones shown by Xiao et al. [8]. If not corrected in time, these process anomalies can lead to reduced yield with consequences in both environmental and profitability terms.

Powder surface monitoring can be carried out using different technologies, each with its own pros and cons. In general, the major concern when it comes to measuring surface topography is resolution, both lateral (in-plane, XY) and vertical (out-of-plane, along Z). Off-axis imaging in either visible or NIR range can be performed using one or more cameras, with lateral resolution ranging from 10 [9] to 290 μm [10] but lack of utility for the topography itself, since it does not natively provide height-resolved images but only bi-dimensional ones. Fringe projection allows mapping of a surface by using a DLP, which projects a light pattern on the target and is acquired by a single or multi-camera system. Several authors tried this technique in powder fusion [3,11,12], but this requires extensive modifications of the build chamber and is very sensitive to quality of the light, reflection phenomena etc. In terms of resolution, values between 7 [3] and 100 μm [12] have been reported in the lateral direction while values below 10 μm have been reported in the vertical one [12]. More complex stereoscopic vision systems have the potential for three-dimensional measurement but are hampered by time-consuming elaboration and require distinct points easily identifiable in multiple images, not available in featureless powder bed surfaces. Finally, another approach is to integrate the sensor directly on the recoater arm, taking advantage of its motion during recoating to qualify the powder bed. Such recoater-mounted sensors include cameras [13] and laser-line scanners [4], and feature comparably easy integration and high resolution, especially laser profilers (Barrett et al. [4] reports a lateral resolution of about 15 and vertical of 0.5 μm). Among all these different monitoring technologies, laser profilometry seems the most interesting one, since it allows quick data evaluation and easy output to the machine controls, fundamental for a successful industrial integration at a comparably low price. Furthermore, in terms of resolution, newer products with respect to what was used by Barrett et al. [4] provide higher resolution both along XY (2.5 μm) and Z (0.3 μm).

Powder layer density is one of the metrics used in the literature [14] to study powder flowability and the associated influence on powder bed fusion processes. Haferkamp et al. [15] demonstrated a lack of correlation between layer density and part quality with a wide range of 316L stainless steel powders characterized by different particle size, however the dynamics of the material-melt pool interactions in metal laser powder bed fusion is drastically different than when using polymer powder. Since preheating is applied in the case of polymer feedstock, the laser-matter interaction happens close to the powder melting point and hence, a reduced thermal shock for the feedstock leads to a more stable process. Vetterli [16] explored powder properties on parts by separating a commercially-available polyamide 12 powder into eleven fractions with varying D50,vol from about 11 to about 60 μm. The main advantage of such an approach is that the macromolecular, thermal, and optical properties of the resulting feedstock are all the same, and only the particle size distribution (PSD) is varied, allowing the study of the isolated influence of PSD on the quality of structures. Among other properties, sintered part density was measured on cubes while powder bed density was obtained with the methodology introduced by Niino and Sato [17], which consists of creating parts made by of a solid shell that traps unsintered powder.

**Figure 1:**
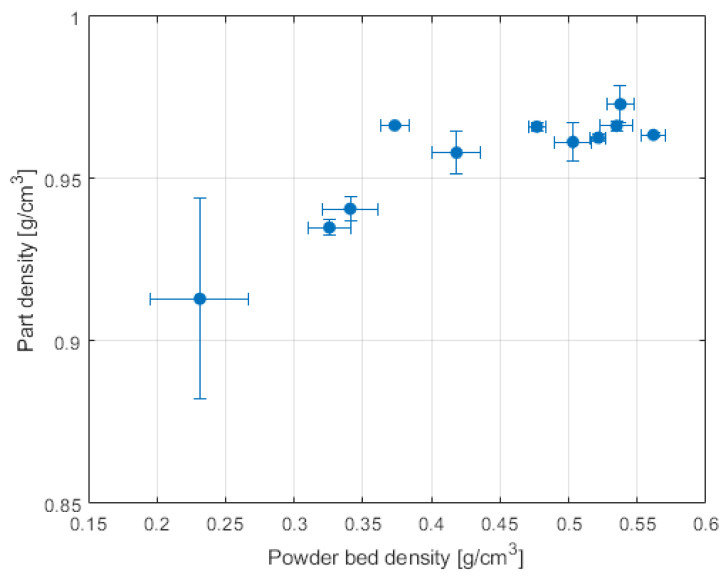
Powder bed density vs. part density for a single powder, data from Vetterli [16].

After the part was removed from the build chamber, the powder was extracted via a (drilled) hole and the trapped powder was weighed with a scale. Since the volume of the cavity is known, the powder bed density was accurately calculated. A correlation between powder bed density and final sintered part density was found and is shown in Figure 1.

Drummer et al. [18] also studied experimentally the influence of several parameters on the density of specimens produced with PBF-LB/P and polyamide 12, and concluded that both the recoating medium (rake vs. roller) and the recoating speed have an effect on final part density. Using a roller, part density is reported to be speed-dependent, while the parts produced using a rake are characterized by a lack of correlation. Schiochet Nasato and Pöschel [19] simulated the effect of different particle shapes for polymer powders, reporting an increase of $R_{q}$ and a decrease of packing density with increasing recoating speed speed. In a more recent article, Nasato et al. [20] modelled with DEM the effect of vibrations applied vertically on the recoater, and obtained once again the same negative correlation between recoating speed and packing density.

**Figure 2:**
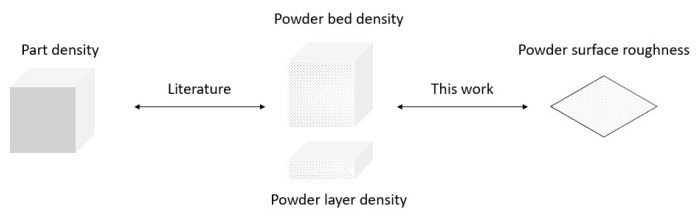
Correlation logic.

The hypothesis of the current work is that the powder layer roughness, as measured with 3D contactless laser-line scanner in a layer-by-layer manner, is correlated with the underlying powder bed density, which is also correlated with part density (Figure 2).

Scope of this effort is to test the robustness of this hypothesis by creating as many combinations of powder surface roughness and powder layer density as possible, utilizing several commercial materials and different recoating speeds. In order to do that, a custom-built powder test bench was used, which mimics the recoating mechanism of a polymer powder bed fusion system and allows effortless substitution of the recoater mechanism as well as variation of additional recoating parameters (recoater speed, layer thickness, etc.). This work aims at providing the foundations for evaluating laser line scanners to advance process monitoring powder bed fusion of polymers as required by the stringent standards for applications in the biomedical, aerospace or automotive industries.

## 2. Materials and Methods

### 2.1. Measuring Setup

**Figure 3:**
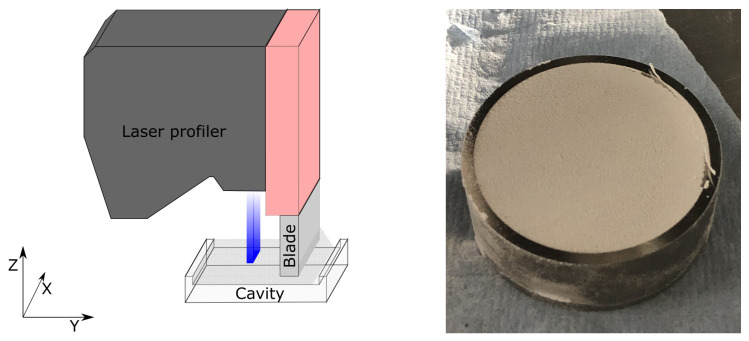
Schematic of the measuring setup (**left**) and filled cavity just before PLD measurement (**right**).

In this work, a Keyence LX-8060 (Keyence, Osaka, Japan) laser line scan profilometer was mounted on a powder test bench, which is specifically designed to recoat single layers of powder in a repeatable manner with adjustable speed and recoating mechanism, mimicking an actual powder bed fusion machine. The profiler features a repeatability in the Z and X axes of 0.4 and 0.5 μm respectively, and a XY pixel size of 10 μm (set by the axis encoder) over a scanning length in the X direction of about 16 mm. Using a steel blade within the test bench, about 12 cubic centimeters of powder were deposited into the metal cavity with a circular shape, a nominal depth of 140 μm and a calculated volume of 177.71 mm^3^ using the procedure developed by Haferkamp et al. [15]. The goal of the cavity is to mimic the actual layer thickness of a powder bed fusion process, and in this specific case, an intuitive correlation between powder bed in a PBF machine and layer density in the test bench can be assumed. In fact, the bed consists of multiple layers deposited one after another. After coating the test bench, the filled cavity is carefully removed and weighed so that the mass of the powder in the single layer can be measured. Then, the so-called powder layer density (PLD) is calculated with Equation (1):

(1) $$PLD=\frac{m_{powder}}{V_{cavity}}\cdot \frac{1}{\rho_{material}}$$

with $m_{powder}$ being the mass of the powder, $V_{cavity}$ the volume of the cavity and $\rho_{material}$ the density of the solidified material according to its datasheet. The resulting PLD value is expressed as percentage and allows to compare materials with different (solidified) density. For every combination of material and recoating speed, PLD was measured three times for statistical purposes. An example of filled cavity is shown in Figure 3 (left). The depth of the resulting powder layer surface was acquired in real-time during the cavity-filling motion by mounting the laser line scanner directly on the recoater arm with the depth captured directly behind it, as depicted in Figure 3 (right).

### 2.2. Materials

A Design of Experiment (DoE) was implemented with five different *v* (50, 75, 100, 150 and 200 mm/s) and eight commercially available materials, as reported in Table 1 in order to create as many combinations as possible of PLD and $S_{q}$. The selection of powders included the most widely adopted feedstock, polyamide 12, provided by two vendors as well as with additives to improve strength and flame retardance. Additionally, polyamide 11 with and without coloring additives from one vendor was included. Polypropylene was included due to interest in sustainable (due to its 100% recyclability) and high-elongation applications and finally thermoplastic polyurethane (TPU) was also added due to superior elasticity and damping. Except for this thermoplastic polyurethane grade, which was still usable for producing parts, all materials are available for purchase. The powders were used in this evaluation as received and without preconditioning. Information about PSD are reported in Table 2, and were obtained using a LS230 laser diffraction device (Beckman Coulter, Brea, CA, USA) with conventional measurements taken on dispersed samples ($0.03{\%}_{wt}$ in ethanol), and the diffraction pattern was evaluated using the Fraunhofer model.

### 2.3. Data Evaluation

**Figure 4:**
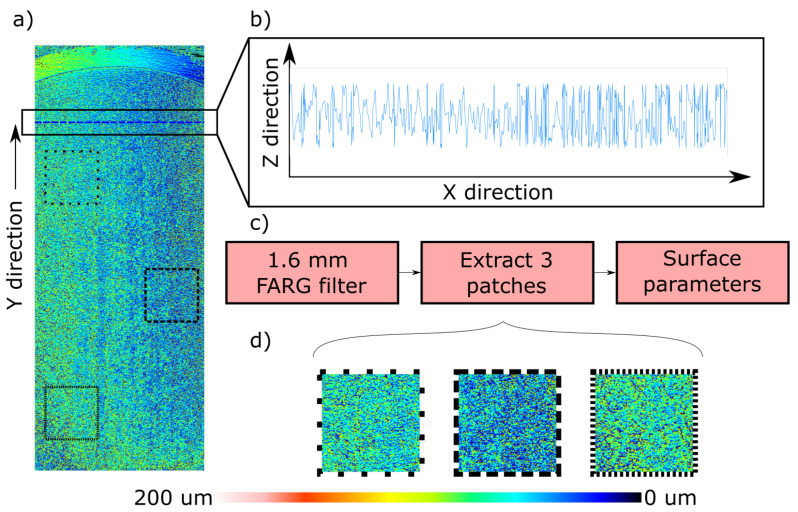
Example of surface map (**a**) with extracted profile (**b**), analysis algorithm (**c**) and extracted patches (**d**).

For each layer recoating, a 40 × 16 mm surface map was captured using the laser profiler that consisted of 4000 × 1600 points with a spacing of 10 μm in both the X and Y axes. An example of such a map is shown in Figure 4a, and a single profile in Figure 4b. Three regions of interest (ROIs, black squares in Figure 4a) were extracted from around the center of the image and treated using the software MountainMaps (Digital Surf, Besançon, France) with the algorithm shown in Figure 4c. For every ROI, the root mean square height $S_{q}$ was calculated according to Equation (2) and the average value for each ROI was used. The decision of using three ROIs instead of the entire surface was based on the possible presence of faulty points, difficult to detect, which would make the evaluation of $S_{q}$ erroneous if used.

(2) $$S_{q}=\sqrt{\frac{1}{A}{\int \int }_{A}Z^{2}\left(x,y\right)\;dx\;dy}$$

The Pearson’s correlation coefficient *r* was used to identify relations between the variables used (*v*, $S_{q}$ and PLD), and was considered statistically significant with p<0.05. This coefficient can be calculated according to Equation (3):

(3) $$r_{x,y}=\frac{cov(x,y)}{\sigma_{x}\sigma_{y}}$$

*r* measures the linear correlation between two sets of data, and its possible values lie between −1 (negative correlation) and +1 (positive correlation), while 0 indicates lack of correlation.

## 3. Results

**Figure 5:**
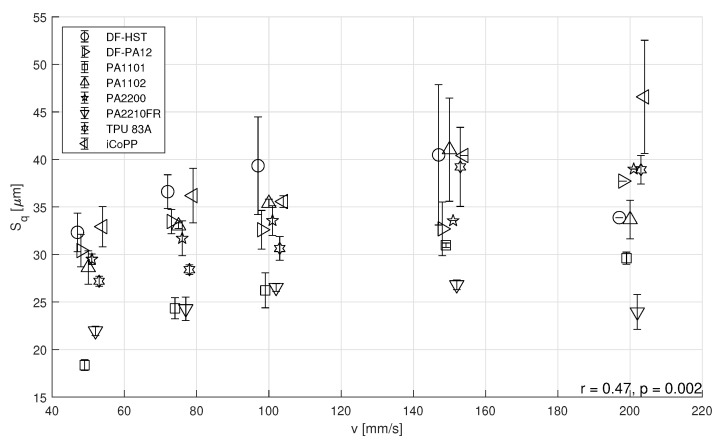
Surface roughness ($S_{q}$) versus recoating speed per material.

Since the laser profiler was mounted on the recoating arm, data acquisition is carried out simultaneously with recoating and thus would not add any delay during processing. Regarding data elaboration, the algorithm takes approximately 10 s per surface map. An alternative approach using Python requires 3 s, and less time would be required if the procedure would be programmed on the control unit of the profiler. Nevertheless, considering a typical layer time of 60 s, data acquisition and elaboration does not add any delay. A positive (r=0.47) and statistically significant (p=0.002) correlation was identified between recoating speed *v* and powder surface roughness $S_{q}$ as depicted in Figure 5. In order to distinguish among overlapping data points, a relative shift has been applied on the x axis, but in reality only five values of *v* have been used, as reported in the previous section.

For industrial applications, maximizing the recoating speed increases productivity as the recoating process interrupts the melting via laser and can constitute a large fraction of time per layer, particularly for large machines and fewer parts [21]. Nevertheless, certain materials are known to have problems with “traditional” recoating systems (smooth roller), and consequently, different approaches have been evaluated including increasing the recoating diameter and roughness in an attempt to provide more energy to the powder for fluidization, an effective solution for materials such as Duraform Flex [22]. Although the average value of $S_{q}$ across all speeds increases significantly (p=0.002), differences among materials are present. Nevertheless, scope of this work is to test the correlation between $S_{q}$ and PLD, not looking at single material or speed, but focusing instead on the general, universal trend. Therefore, there exists an increase of surface roughness with increasing speed, confirming the outcome of Schiochet Nasato and Pöschel [19]. Nonetheless, in the present work the results were experimentally obtained using very different feedstock in terms of chemical composition, PSD, production method and shape. Powder shape did not appear to play a critical role and highly-spherical powders (such as iCoPP [22]) seem as affected by recoating speed as cryogenically-milled polymers (such as TPU). Briefly focusing on DF-PA12, its composite DF-HST (which contains mineral fibers [22]) and PA2200, which are polyamides 12 obtained through dissolution-precipitation and characterized by particles exhibiting a potato shape (smooth and elongated), it seems that almost no influence is exerted on powder surface roughness by recoating speed. This material class represents the state of the art for polymer powder bed fusion and the lack of variability in the $S_{q}$ parameter across the different speeds appears to be a consequence of the high degree of powder optimization. However, it is not possible to obtain smoother powder layers even at low speeds. As other authors have already observed [19], elongated particles might behave similarly or even better than spheres at low recoating speeds as these powders tend to align themselves in the direction of the recoating flow. According to the same work, in DEM simulations that implemented particles with different shapes, the favorable alignment happens up until a speed of 250 mm/s, which was not possible to test in the present effort due to excessive accelerations required over a short distance. Nevertheless, large machines such as the EOS P7xx series, which feature a platform size of 700 mm, or the Farsoon HT1001P, with a 1000 mm long build platform, can provide higher recoating speeds and would benefit from the availability of well-behaved materials. Interestingly, spherical powders (i.e., iCoPP) showed the highest degree of correlation between $S_{q}$ and *v*, exhibiting a r=0.97 with a p=0.004: this means that this specific feedstock changes its surface roughness upon spreading the most with increasing speed

**Figure 6:**
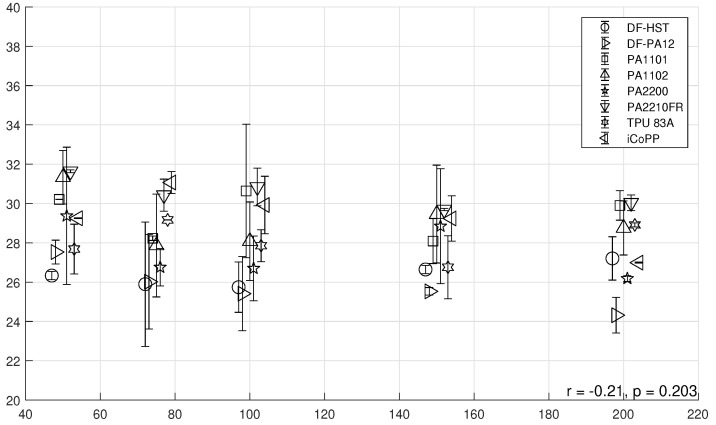
Powder layer density PLD vs. recoating speed *v*.

Powder layer density (PLD) was measured against recoating speed for all materials, and the results are given in Figure 6, again with a small visual shift of the data points along the x axis to help visualization.

In this case, the same correlation between recoating speed and powder layer density cannot be observed (r=−0.21 but not statistically significant), although a minimal decrease can be seen from 50 to 100 mm/s in almost all materials. This lack of correlation might be related to a pronounced wall effect, since the cavity depth is only about 2.5 times larger than the D50 of the powder, and this limits its accommodation. Furthermore, in order to precisely measure the PLD, a metal surface with roughly the same roughness as the powder is used as substrate, and hence the behavior of the material might be different compared to “powder on powder” deposition of the real processing. According to Schiochet Nasato and Pöschel [19], powder layer density should decrease with increasing recoating speed, but this was not observed significantly in this dataset. The experimental evaluation of the powder layer density seems to be a weak point, and the methodology proposed by Niino and Sato [17], which features laser sintering of hollow parts and subsequent measurement of the bed density by weighting the trapped powder, is more robust and will be the focus of future work. Nevertheless, the material that exhibit the highest decline of PLD with increasing *v* is DF-PA12, with a drop of the mean value of PLD from almost 27.5% to 24.3%. This negative correlation (r=−0.89) is also statistically significant (p=0.04).

**Figure 7:**
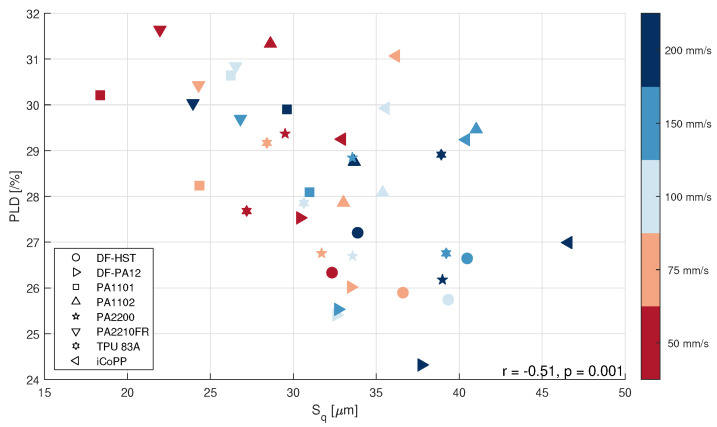
Powder layer density (PLD) vs. powder surface roughness ($S_{q}$) for all materials and speeds.

A statistically-significant (p=0.001) Pearson’s correlation coefficient r=−0.51 was found between PLD and $S_{q}$, as can be seen from Figure 7 where error bars were omitted for simplicity.

**Figure 8:**
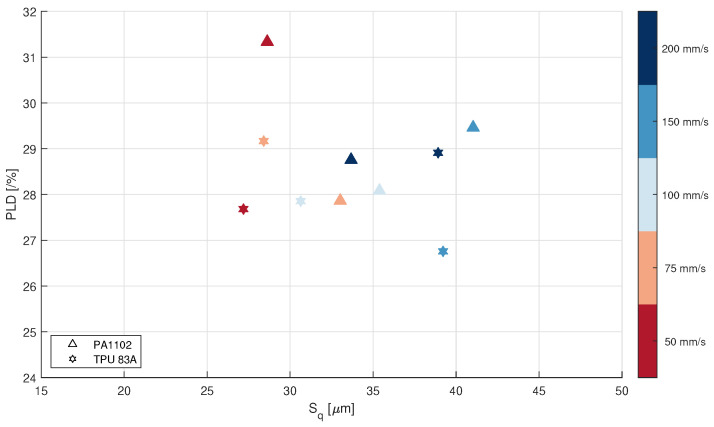
PLD vs. $S_{q}$ for PA1102 and TPU 83A (different PSD).

The material exhibiting the highest correlation between PLD and $S_{q}$ is again DF-PA12 (r=−0.88, p=0.04). Finally, a comparison between PA1102 and TPU 83A can be observed in Figure 8: these two materials represent two very different PSDs ($D_{50}=50\;\mu$m and $D_{50}=80\;\mu$m, respectively) and it seems that their behavior is different, with a sharper decrease of the PLD value with increasing $S_{q}$ for PA1102 compared to TPU. So, it seems that the PSD influences somehow both $S_{q}$ and PLD, but more experiments are needed to exclude the influence of other (uncontrolled) variables.

## 4. Conclusions and Outlook

In situ process monitoring is extremely important to increase the industrial adoption of polymer powder bed fusion. Among the different aspects that can be gauged, the powder bed is certainly one of the most critical yet complex to evaluate. Laser profilometry may be a reasonable compromise between cost and quality. Moreover, data elaboration can be implemented relatively quickly and hence this technology is well suited for integration into industrial-grade powder bed fusion machines. In this work, the relationship between powder layer density (PLD) and surface roughness ($S_{q}$) has been explored for eight different polymer feedstocks at five different recoating speeds, which constitute a relatively large dataset useful for obtaining statistically significant results. $S_{q}$ has been demonstrated to increase with increasing recoating speed, and thus faster recoating leads to rougher powder beds for the majority of materials tested. At the same time, an increase of $S_{q}$ can be related in a statistically-significant manner to a decrease in powder layer density, one of the metrics used to evaluate powder bed density, which has been previously demonstrated as a proxy for final part density, and ultimately, for mechanical performances. Outlook of the present work is the integration of this measurement technology in a powder bed fusion machine, where it would be possible to study the temperature influence on the packing efficiency of the powder, since it affects the relative importance of the cohesive forces among particles, and this justifies the need for an effective process monitor tool even further.

## Figures and Tables

**Table 1:** Materials list.

Producer	Commercial Name (Short Name)	Polymer Type
3D Systems	Duraform PA12 (DF-PA12)	Polyamide 12
3D Systems	Duraform HST (DF-HST)	Polyamide 12 + 11% wollastonite fibers
EOS	PA1101	Polyamide 11
EOS	PA1102	Polyamide 11 + carbon black
EOS	PA2200	Polyamide 12
EOS	PA2210FR	Polyamide 12 + halogen-free flame retardant additives
research grade	TPU 83A	Thermoplastic polyurethane
inspire irpd	iCoPP	Polypropylene

**Table 2:** Particle size distribution, all values are in μm.

Material	$D_{10}$	$D_{50}$	$D_{90}$
DF-PA12	28	56	82
DF-HST	24	57	83
PA1101	21	51	78
PA1102	20	50	73
PA2200	37	59	84
PA2210FR	24	59	82
TPU 83A	37	80	120
iCoPP	35	63	123
